# Heat Shock Protein-90 Inhibitors Enhance Antigen Expression on Melanomas and Increase T Cell Recognition of Tumor Cells

**DOI:** 10.1371/journal.pone.0114506

**Published:** 2014-12-12

**Authors:** Timothy J. Haggerty, Ian S. Dunn, Lenora B. Rose, Estelle E. Newton, Franco Pandolfi, James T. Kurnick

**Affiliations:** 1 CytoCure LLC, Suite 430C, 100 Cummings Center, Beverly, MA, United States of America; 2 Department of Pathology, Massachusetts General Hospital, Harvard Medical School, Boston, MA, United States of America; 3 Department of Internal Medicine, Catholic University, Rome, Italy; MRC National Institute for Medical Research, United Kingdom

## Abstract

In an effort to enhance antigen-specific T cell recognition of cancer cells, we have examined numerous modulators of antigen-expression. In this report we demonstrate that twelve different Hsp90 inhibitors (iHsp90) share the ability to increase the expression of differentiation antigens and MHC Class I antigens. These iHsp90 are active in several molecular and cellular assays on a series of tumor cell lines, including eleven human melanomas, a murine B16 melanoma, and two human glioma-derived cell lines. Intra-cytoplasmic antibody staining showed that all of the tested iHsp90 increased expression of the melanocyte differentiation antigens Melan-A/MART-1, gp100, and TRP-2, as well as MHC Class I. The gliomas showed enhanced gp100 and MHC staining. Quantitative analysis of mRNA levels showed a parallel increase in message transcription, and a reporter assay shows induction of promoter activity for Melan-A/MART-1 gene. In addition, iHsp90 increased recognition of tumor cells by T cells specific for Melan-A/MART-1. In contrast to direct Hsp90 client proteins, the increased levels of full-length differentiation antigens that result from iHsp90 treatment are most likely the result of transcriptional activation of their encoding genes. In combination, these results suggest that iHsp90 improve recognition of tumor cells by T cells specific for a melanoma-associated antigen as a result of increasing the expressed intracellular antigen pool available for processing and presentation by MHC Class I, along with increased levels of MHC Class I itself. As these Hsp90 inhibitors do not interfere with T cell function, they could have potential for use in immunotherapy of cancer.

## Introduction

While there is widespread interest in mobilizing anti-tumor immunity, there remain barriers to immunotherapy [1] [2]. Therapeutic successes have been achieved through adoptive transfer of both CD8+ tumor-reactive cytotoxic T cells (CTL) [3] and CD4+ tumor infiltrating lymphocytes (TIL) [3], [4]. Recently, there has been significant progress using adoptive transfer of cells that are programmed to express Chimeric Antigen Receptors (CAR), allowing for therapy with highly defined effector populations [5]. In addition, there is increasing awareness that CD4+ regulatory T cells (Tregs) play an important role in inhibiting anti-tumor immunity [6].

However, even when tumor-specific T cells are enriched within tumor sites, this immune response does not necessarily lead to control of tumor growth [6]. Notably, generating effective immunity can be limited by numerous suppressive factors in the tumor microenvironment, including antigen regulatory factors produced by the tumor cells [7]. Some of the down-regulatory effects on the host immune response have been inhibited therapeutically via neutralization of Treg cells, blockade of the PD-1/PD-L pathway, or inhibition of myeloid-based immunosuppressive molecules [8], including targeting of T cell activation checkpoints such as CTLA-4, but such therapies may be limited by serious side effects [9].

In addition to effects on immune cells, heterogeneity within the tumor itself also plays an important role in limiting the efficacy of the immune response. This communication focuses on approaches to overcoming the loss of tumor antigen expression [7], [10]–[12], to address this route of tumor escape from T cell-mediated immunity [13]. While antigen loss may be the result of ongoing immune pressures, including immune editing [14], we have demonstrated that there are several ways to restore antigen expression, including MAP-kinase (MAPK)- inhibitors [11], Interferon-beta (IFN-β) [10], topoisomerase inhibitors [15], and most recently iHsp90 [16].

Based on a screen for agents that enhance T cell recognition of Melan-A/MART-1, the iHsp90 17-Allylamino-17-demethoxygeldanamycin (17-AAG) was recognized as a potent stimulus of melanoma antigen expression [16]. By inhibiting Hsp90, 17-AAG causes the destabilization of the products of several mutant oncogenes, including BRAF, CRAF and NRAS [17]. Through its role in regulating the conformation, stability and function of several key oncogenic client proteins, Hsp90 is essential in maintaining malignant transformation and in increasing the survival, growth, and invasive potential of cancer cells, including melanomas [18] [19]. Several members of this drug class have been tested in human clinical trials [20], and while the drugs may slow tumor growth, to date none have succeeded as single agents [21].

Notably, iHsp90s have been shown to increase T cell recognition of both Her-2 [22] and EphA2 [23] antigens. Both of these onco-proteins are known client proteins of Hsp90, and while the levels of intracellular expression of these antigens were *decreased* after Hsp90 treatment, the enhanced CTL-recognition of the treated tumor cells was attributed to increased turnover of the proteins, combined with augmented peptide presentation on MHC molecules. In contrast, evidence suggests that the differentiation antigens and MHC Class I proteins that increase in response to iHsp90 are not Hsp90 client proteins, and iHsp90 treatments result in enhanced T cell recognition as a result of increased expression of the actual target proteins.

As there are dozens of new iHsp90 being developed, the possibility that this class of drugs could be used therapeutically offers a novel approach to combination immunotherapy. Studying an extended tumor cell line panel (including 11 different human melanoma cell lines with differential BRAF or NRAS mutations, as well as a murine melanoma cell line and two human glioma cell lines) provided evidence that iHsp90 treatment may be effective on different neural crest-derived tumors. Importantly, the iHsp90s are able to enhance antigen expression on the tumor cells while retaining T-cell function [16], suggesting that this class of drugs could be utilized in immunotherapy to enhance tumor targeting.

## Materials and Methods

### Ethics Statement

The studies reported involve human cell lines that were either obtained from a commercial source (ATCC, Manassas, VA), or from previously reported cell lines obtained from the Massachusetts General Hospital following guidelines approved by the IRB of this hospital. Cell lines obtained from humans were approved by the Human Studies Committee of the Massachusetts General Hospital at the time of their establishment.

### Cell culture, and iHsp90

Culture conditions and the cell lines have been previously described [12]. The B16-BL6 murine melanoma cell line was provided by Dr. Andrew Hurwitz [24]. Human cell lines were obtained from the ATCC, (Manassas, VA) (including MALME-M3, MM96L, A375, 453A, MM455, Roth, H59-44T, Mel-Juso, JURKAT), or from a patient at the Massashusetts General Hospital (MU89 and MU-X (derived from MU89 as previously described [6], [11]). Construction and culture of the J.RT3-T3.5 cell line expressing a Melan-A/MART-1 specific TCR has been described [15]. IFN-β-1a (Avonex) was obtained from Biogen-Idec (Cambridge, MA). The iHsp90s were obtained from the following sources: Radicicol (A.G. Scientific, San Diego, CA); Novobiocin (BioMol, Plymouth Meeting, PA); 17-DMAG (LC laboratories, Woburn, MA); 17-AEP (InVivoGen, San Diego, CA); Rifabutin, PU-H71, and 17-AAG (Sigma, St. Louis, MO); Gedunin, CCT018159, and celastrol (Tocris, Ellisville, MO); and NVP-AUY922 and BIIB021 (Selleck Chemicals, Houston, TX).

### EGFP reporter cell line

The generation and application of Melan-A/MART-1 promoter-linked EGFP reporter cells has been previously described [10]. Stable transfectants were generated in the low-antigen cell line A375 and the high-antigen line MM96L+ and MU89.

### Measurement of cell growth

Cell growth was measured using the WST reagent system from Roche (Indianapolis, IN). In brief, 2 × 10^3^ cells were plated in culture plates. The time zero absorbance (T_o_) of the cells was measured at 450 nm 24 hours after plating. Following 72 h of triplicate well culture with test agents, WST was added to each well and after 1 h the hydrolysis of the WST was read (T_x_) in a plate reader at 450 nm. Untreated control cells were measured (Con) to establish 100 percent growth. Wells were normalized by subtracting the 655 nm background reading. % Growth =  100× (T_o_ – T_x_)/(Con-T_x_).

### Treatment of cells with iHsp90

Cells were plated at a density of 1×10^5^ in 1 ml of medium in a 24-well plate and cultured as indicated in results. Cells were collected by trypsinization for assays described below.

### Flow cytometry

Intracellular staining and cytometric analyses of cytoplasmic Melan-A/MART-1, and gp100 expression were performed as described [12]. Cells were fixed, permeabilized and stained with monoclonal antibodies to Melanocyote Antigens The following Antibodies were used as described [10]: Melan-A/MART-1 (Vector Labs, Burlingame, CA); gp100/HMB45 (DakoCytomation, Carpinteria, CA) using a goat anti-mouse FITC-conjugated secondary antibody (Invitrogen, Frederick, MD). The level of EGFP was determined by cytometry of unfixed cells washed and re-suspended in PBS. Surface staining of human MHC Class I was performed, on ice, using the antibody W6/32 on cells harvested with Cell Stripper (MediaTech Inc., Manassas, VA). Mouse anti H-2 D^b^ (Clone 28-8-6, BioLegend, San Diego, CA) was used to stain for MHC Class I antigens on B16 melanoma cells.

### RNA preparation and Quantitative PCR (qPCR)

To compare untreated and iHsp90-treated cells for changes in gp100 (pmel17) mRNA levels, total RNA was extracted and reverse transcription to cDNA was performed as previously described (8). qPCR was carried out using SYBR Green detection [25] in an Mx3000P thermocycler with analysis performed using the proprietary MxPro Software (Agilent Technologies, Santa Clara, CA). Each reaction contained 25 ng of cDNA template, 10 µM of each primer and 10 µl of 2X SYBR Green PCR Master Mix (Life Technologies, Grand Island, NY). Triplicate reaction replicates were amplified for 40 cycles (95°C for 15 seconds and at 60°C for 1 minute) after initial heat activation at 95°C for 10 minutes. The fold increase of mouse gp100 cDNA in iHSP90 treated *vs*. untreated cells was determined relative to glyceraldehyde 3-phosphate dehydrogenase (GADPH), and β-Actin. Fold increase  =  2^-ΔΔCt^ where ΔΔ*C*_t_  =  (*C*_t_GOI - *C*_t_Reference Gene) Treated - (C_t_GOI - C_t_Reference Gene)Untreated [26]. (GOI =  gene of interest =  gp100; *C_t_*  =  threshold cycle number).

Primers used for each gene are as follows:

**gp100 (pmel17)**: Forward-ACCTGGAACCACATCTAGGC;

Reverse-TCCAGAGGGCTGTGTAGTTG; product  =  100 bp;

**GADPH**: Forward- TGCCCAGAACATCATCCCTG,

Reverse-GACGGACACATTGGGGGTAG; product  =  121 bp;

β **-Actin**: Forward-CCACCATGTACCCAGGCATT,

Reverse-CGGACTCATCGTACTCCTGC; product  =  189 bp.

### Western blot analysis

Cell lysates were prepared using RIPA buffer (Santa Cruz, CA) containing protease and phosphatase inhibitors. Protein concentrations were determined using Bradford assay (BioRad, Hercules, CA). Equal amounts of protein were loaded in each well for SDS-PAGE analysis. Proteins were transferred to PVDF membranes (Pierce Biotechnology, Rockford, IL). Blocking and both primary and secondary antibody incubations were performed using Starting Block (Pierce). Blots were washed with TBS with 0.5% tween 20. Primary antibodies used: BRAF H-145 (Santa Cruz, CA), Phos-MEK and Phos-ERK (Cell Signaling Technologies, Danvers, MA) β-actin (Sigma, St. Louis, MO). Goat anti-rabbit HRP conjugated secondary antibody (Pierce, Rockford, IL). Chemiluminescence was performed using the Femto kit (Pierce, Rockford, IL).

### Assays of CD8+T lymphocytes

Primary CD8+ T lymphocytes were obtained as previously described [15] and cultured in medium supplemented with 200 IU/ml recombinant IL-2 (Proleukin, Cetus, Emeryville, CA). For transduction of the Melan-A/MART-1 specific TCR, 1 × 10^5^ stimulated primary CD8+ cells were incubated with lentiviral vector at a multiplicity of infection (MOI) of 10. Cells were grown for two days after lentiviral infection and stained with tetramer [15]. The CD8+ T cells were further propagated for 2 weeks in medium supplemented with IL-2, prior to use in cellular assays described below.

### Assay for T cell activation by tumor cells

Melanoma cells were treated with antigen-modulating agents, (iHsp90 or IFN-β) for 3 days prior to counting and plating for the T cell co-culture assay. 5×10^4^ tumor cells were co-cultured overnight (16 to 24 hours) with 2.5×10^4^ Jurkat T cells (stably transduced to express a TCR specific for Melan-A/MART-1) prior to removal of supernatant fluids for assay of cytokine production as previously described [16]. To assess antigen-specific T cell responses, 3.33 µg/ml Melan-A/MART-1 peptide was added to the co-culture. The sequence of the Melan-A/MART-1 peptide used is _26_ELAGIGILTV_35_ (A27L from wild-type).

### Cytokine ELISA

Protocols for ELISA evaluation of IL-2 and IFN-γ followed the manufacturer's recommendations as previously described [16]. The HRP color reaction proceeded for 15 min before being stopped with 2N H_2_SO_4_. The absorbance at 450 nm was read in a BioRad 3550 plate reader. IL-2 levels for experimental samples (pg/ml) were calculated from the standard curve.

## Results

### (1) Multiple iHsp90s are active on diverse tumors and antigens

We tested an expanded group of iHsp90s including 17-AAG derivatives (such as 17-AEP), as well as structurally distinctive compounds, such as PU-H71 and CCT018159. As shown in Table 1, these additional iHsp90 are each active on a variety of melanoma cell lines with different levels of antigen expression, including MALME, MU-89, A375, MU-X, a murine melanoma cell line (B16), and two glioma cell lines (U-87MG and U-118MG). The melanomas all express varying levels of differentiation antigens Melan-A/MART-1, gp100 and TRP-2, as well as the MHC Class I antigen (as evidenced by staining with W6/32 antibody) that is required for T cell recognition of the tumor cells. Each of these antigens is enhanced by all of the iHsp90, although the melanomas expressing higher levels of antigen (MALME and MU89) prior to treatment respond to a greater extent than the low-antigen tumors (A375 and MU-X). The murine melanoma, B16, can be stained with the gp100 and TRP-2 antibodies (both are raised against human proteins, but cross-react with their murine homologs), but the Melan-A/MART-1 antibody does not react with B16 melanoma. The antigen levels were compared as mean fluorescence in Flow Cytometric analyses of at least 1000 viable cells per sample. Because of the sample size with normally distributed fluorescence the t-test statistical analysis was highly significant (p<0.0001) in almost all samples. Of the data in Table 1, all samples reaching a ratio of 1.2 or greater reached this level of significance. Of note, the iHsp90 stimulated statistically significant increases in every combination of cell and antigen with the exception of CCT018159 on the U-87 MG glioma MHC Class I antigen expression, and PU-H71 failed to increase TRP-2 on MUX cells. In contrast, CCT018159 was highly effective at induction of MHC Class I antigen on the murine melanoma (6.8 fold induction), and PU-H71 induced strong increases in TRP-2 in these same B16 murine melanoma cells, indicating differential sensitivity of different cell lines to the various iHsp90s. All cell lines were tested in a minimum of 3 different experiments with the panel of iHsp90s shown, and some cell lines were tested more than 10 times, with the data from these repeated studies supporting the data shown in Table 1.

**Table 1:** Effect of Hsp90 Inhbitors on differentiation antigens and MHC Class I.

Cell Line	Treatment^a^	µg/ml^b^	MART-1^c^	gp100^c^	TRP-2^c^	Class I^d^
MALME-3M	Control		117	227	106	44
(+)	IFN-beta		130 (1.1)	534 (2.4)	179 (1.7)	194 (4.4)
	17-AEP	1.0	205 (1.8)	839 (3.7)	256 (2.4)	105 (2.4)
	PU-H71	0.3	226 (1.9)	511 (2.3)	279 (2.6)	137 (3.1)
	CCT018159	10.0	291 (2.5)	823 (3.6)	222 (2.1)	72 (1.6)
MU89	Control		33	123	92	18
(+)	IFN-beta		59 (1.8)	140 (1.1)	150 (1.6)	51 (2.8)
	17-AEP	1.0	130 (3.9)	342 (2.8)	144 (1.6)	54 (3.0)
	PU-H71	0.3	147 (4.5)	381 (3.1)	214 (2.3)	66 (3.7)
	CCT018159	10.0	99 (3.0)	273 (2.2)	252 (2.7)	75 (4.2)
A375	Control		6	15	16	36
(-)	IFN-beta		8 (1.3)	17 (1.1)	24 (1.5)	84 (2.3)
	17-AEP	0.5	11 (1.8)	22 (1.5)	31 (1.9)	67 (1.9)
	PU-H71	0.15	11 (1.8)	24 (1.6)	26 (1.6)	71 (2.0)
	CCT018159	2.5	13 (2.2)	32 (2.1)	37 (2.3)	70 (1.9)
MUX	Control		13	26	41	104
(-)	IFN-beta		18 (1.4)	46 (1.8)	55 (1.3)	158 (1.5)
	17-AEP	1.0	19 (1.5)	34 (1.3)	48 (1.2)	219 (2.1)
	PU-H71	0.3	19 (1.5)	32 (1.2)	41 (1.0)	212 (2.0)
	CCT018159	10.0	22 (1.7)	40 (1.5)	40 (1.0)	174 (1.7)
U-118 MG	Control		n.a.	45	n.d.	50
(glioma)	IFN-beta		n.a.	49 (1.1)	n.d.	132 (2.6)
	17-AEP	1.0	n.a.	61 (1.4)	n.d.	117 (2.3)
	PU-H71	0.3	n.a.	63 (1.9)	n.d.	132 (2.6)
	CCT018159	10.0	n.a.	83 (1.4)	n.d.	101 (2.0)
U-87 MG	Control		n.a.	26	n.d.	27
(glioma)	IFN-beta		n.a.	36 (1.4)	n.d.	73 (2.7)
	17-AEP	0.5	n.a.	51 (2.0)	n.d.	66 (2.4)
	PU-H71	0.3	n.a.	66 (2.7)	n.d.	53 (2.0)
	CCT018159	5.0	n.a.	71 (2.5)	n.d.	28 (1.0)
B16	Control		n.a.	80	94	2
(murine	17-AEP	0.5	n.a.	420 (5.3)	288 (3.1)	13 (5.4)
melanoma)	PU-H71	0.15	n.a.	595 (7.4)	360 (3.8)	14 (6.0)
	CCT018159	2.5	n.a.	414 (5.2)	364 (3.9)	16 (6.8)

a Cells were untreated (control) or treated with 5000 Units/ml of IFN-beta, or with the Hsp90 inhibitors as indicated for 3 days.

b Dose indicated is optimal dose for antigen increase

c Number represents geometric mean of intracellular staining with an antibody to Melan-A/MART-1, gp100 or TRP-2 of live gated cells. Number is parenthesis is fold increase relative to untreated control.

d Number represents geometric mean of surface staining with the MHC Class I antibody W6/32 (or H2kb for B16) of live gated cells.

Antigen status of human melanoma cell lines: (+)  =  Melan-A/MART-1and gp100 high (-) = Melan-A/MART-1and gp100 low.

n.a.  =  not applicable, glioma do not express Melan-A/MART-1, and human Melan-A/MART-1 antibody did not cross react with murine Melan-A/MART-1

n.d.  =  not determined; these cells were not stained with the TRP-2 antibody.

Melanomas and gliomas are of neural crest origin, and gliomas are known to express gp100, but not most of the other melanocyte differentiation antigens. Thus, we tested the glioma cell lines for induction of gp100 and MHC class I expression and noted that glioma-derived cells can also be enhanced by iHsp90 treatment (Table 1).

### (2) iHsp90 activity on melanomas with differing mutant BRAF status

Previous work has demonstrated that iHsp90 can modulate cellular levels of BRAF and NRAS [17], [27]. Several cell lines expressing wild-type (WT) or mutant (M) alleles of these genes were evaluated, to determine if there is a relationship between levels of iHsp90-mediated antigen up-regulation and BRAF and NRAS mutational status [11]. The highest responses were observed for cells heterozygous for BRAF mutation and WT for NRAS (Table 2); activating mutations in these genes appear to be mutually exclusive [28]. Of note, the iHsp90 increased antigens in both types of cell lines (those with mutant NRAS and WT BRAF, as well as cells expressing WT NRAS and mutant BRAF), indicating that the effects of iHsp90 are not limited to the mutant status of either BRAF or NRAS genes.

**Table 2:** Effect of Hsp90 inhibitors on melanocyte differentiation antigens.

BRAF^a^	NRAS	Cell Line	Treatment^b^	µg/ml^c^	MART-1^d^	HMB45^d^
WT	M/M	Mel-Juso	17-AEP	0.50	1.75	1.81
		(-/+)	CCT018159	5.00	1.17	1.12
			PU-H71	0.15	1.41	1.99
		Roth	17-AEP	0.50	nd	1.89
		(-/+)	CCT018159	2.50	nd	1.95
			PU-H71	0.25	nd	2.97
		H59-44T	17-AEP	0.50	2.09	1.91
		(+)	CCT018159	5.00	3.90	3.77
			PU-H71	0.15	3.30	3.59
M/WT	WT	MU89	17-AEP	0.50	3.61	3.26
		(+)	CCT018159	10.00	3.22	3.92
			PU-H71	0.30	3.42	3.57
		MALME-3M	17-AEP	0.25	2.34	2.25
		(+)	CCT018159	5.00	2.48	3.63
			PU-H71	0.30	1.83	2.25
		453A	17-AEP	1.00	2.29	3.78
		(+)	CCT018159	10.00	3.29	3.38
			PU-H71	0.30	3.22	3.09
		MM96L+	17-AEP	1.00	1.79	0.92
		(+)	CCT018159	5.00	3.04	1.81
			PU-H71	0.30	2.59	1.78
		MM455	17-AEP	0.50	nd	1.39
		(-/+)	CCT018159	2.50	nd	1.16
			PU-H71	0.25	nd	1.50
M/M	WT	MUX	17-AEP	0.50	1.55	1.75
		(-)	CCT018159	5.00	1.64	1.89
			PU-H71	0.30	1.54	1.59
		MM96L-	17-AEP		nd	nd
		(-)	CCT018159	5.00	1.28	nd
			PU-H71	0.15	1.82	nd
		A375	17-AEP	0.50	1.46	1.47
		(-)	CCT018159	2.50	2.22	2.22
			PU-H71	0.15	1.80	1.86

a WT  =  homozygous WT, M/WT  =  heterozygous WT, M/M  =  homozygous mutant.

b Cells were treated with the indicated concentration of Hsp90 inhibitor for 3 days before intracellular staining.

c Dose indicated is optimal dose for antigen increase

d Level of induction of indicated melanocyte proteins relative to untreated control cells.

nd  =  not determined.

+  =  antigen-positive; +/−  =  gp100 positive/Melan-A/MART-1 negative; -  =  antigen-negative

### (3) Characterization of iHsp90 Treatment of Melanoma Cells

#### (A) Effects of iHsp90 on a Melan-A/MART-1 Expression Reporter

**Figure 1:**
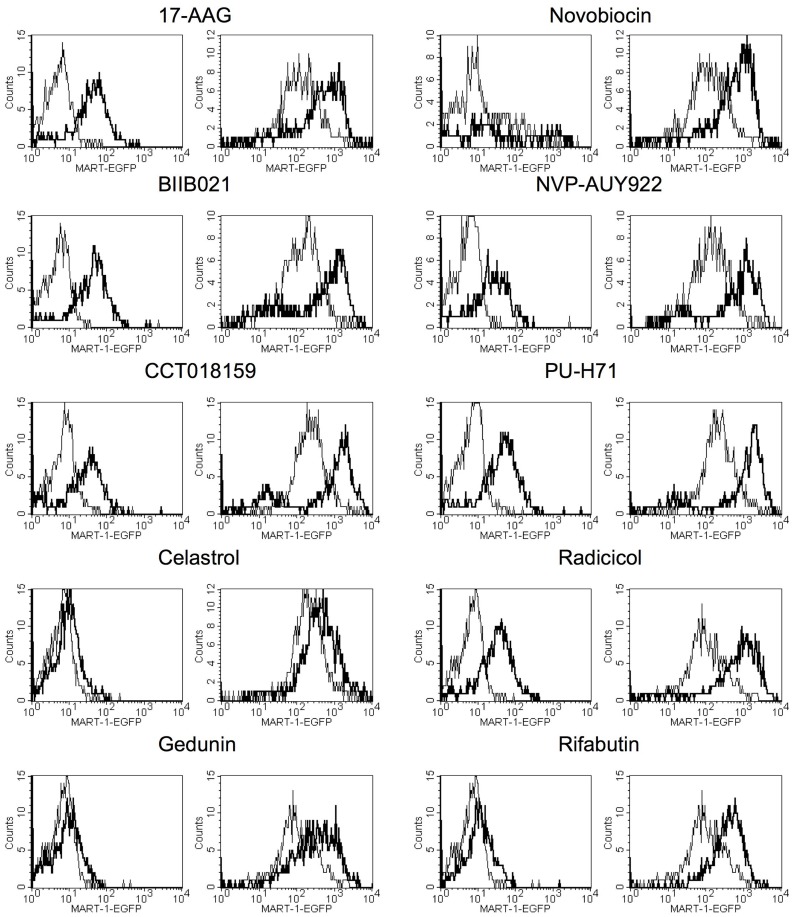
Effect of Hsp90 Inhibitors on Melan-A/MART-1 promoter. Data shown are flow cytometry-generated histograms of EGFP production in reporter cell lines with EGFP linked to the Melan-A/MART-1 promoter. In each histogram, the thin line curve represents the untreated control, and bold line is Hsp90 inhibitor treated cells. In each case, the reporter cells were treated for three days prior to assessing EGFP-related fluorescence. Data are from one representative experiment. The first and third columns are low antigen A375 cells and the second and fourth columns are high antigen-expressing MM96L+ cells. Doses of Hsp90 inhibitor used are listed in Table 1.

**Figure 2:**
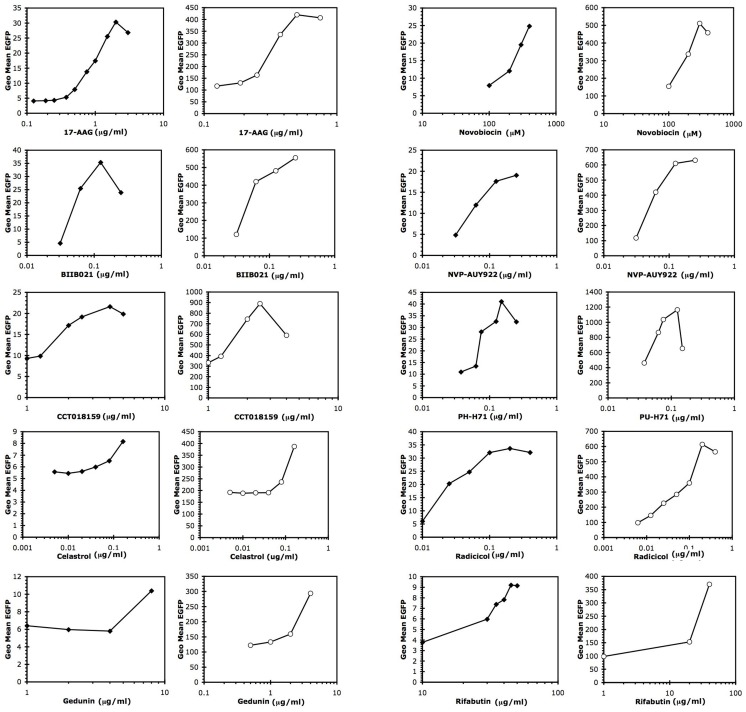
Dose response curves for various Hsp90 inhibitors. As in Fig.1, reporter cells expressing EGFP-linked to the Melan-AMART-1 promoter were treated with a series of Hsp90 inhibitors. Filled diamonds: A375 antigen low Melan-A/MART-1 promoter EGFP reporter cell line. Open circles: MM96L+, high antigen-expressing Melan-A/MART-1 promoter EGFP reporter cell line. Data are from one representative experiment.

Since antigen induction by 17-AAG is accompanied by enhanced reporter gene activity driven by the Melan-A/MART-1 promoter [16], we tested promoter-driving activity of 12 different iHsp90. The tested inhibitors included 7 compounds that bind to the amino-terminal ATP-binding region of Hsp90 [29], while 3 of the inhibitors, (gedunin, celastrol, and novobiocin), do not bind to this ATP-binding site and manifest their activity via distinct mechanisms [30], [31]. Similar relative levels of EGFP induction were observed after treatment with either class of iHsp90 (Table 3). All of the iHsp90 increased EGFP, both in a cell line with low intrinsic levels of Melan-A/MART-1 (A375), and in a cell line with higher levels of endogenous Melan-A/MART-1 (MM96L+) (Fig. 1). These results are consistent with the hypothesis that the effect of iHsp90 on antigen levels operates through transcriptional up-regulation. Optimal doses were determined using dose response curves (Fig. 2).

**Table 3:** Effect of Hsp90 inhibitors on Melan-A/MART-1 promoter-driven EGFP reporter.

Hsp90		A375		MM96L+		
Binding	Treatment^a^	µg/ml^b^	EGFP^c^	µg/ml	EGFP	Ref.
	Control	0.00	6.0	0.00	98.2	
N-term	17-AAG	2.00	42.0	0.50	347.2	[53]
ATP	17-AEP	0.50	16.0	0.13	248.3	[54]
	17-DMAG	0.50	9.4	0.50	644.8	[54]
	PU-H71	0.15	39.5	0.13	541.9	[29]
	CCT018159	4.00	20.0	2.50	389.6	[55]
	Radicicol	0.10	32.1	0.20	614.3	[56]
	Rifabutin	40.00	10.9	40.00	370.1	
	BIIB021	0.13	46.2	0.25	449.8	[57]
	NVP-AUY922	0.25	23.7	0.25	528.7	[58]
cdc37 site	Gedunin	8.00	10.4	4.00	294.0	[59]
	Celastrol	0.20	14.0	0.20	466.3	[31]
C-term ATP	Novobiocin^d^	400.00	21.8	300.00	470.8	[30]

a Cell were treated for 3 days.

b Dose indicated is optimal dose for antigen increase

c Geometric mean of EGFP flow histogram.

d Concenteration of Novobiocin in µM

#### (Aii) Effects of iHsp90 on *Pmel 17* (gp100) Expression in Murine B16 Melanoma

To determine if increased levels of intracellular protein are accompanied by corresponding increases in mRNA transcripts, murine B16 melanoma cells were stimulated for 3 days with CCT018159, followed by parallel protein staining and quantitative PCR (qPCR) analysis. The flow cytometry data demonstrated 4.6 fold increase in the expression of gp100 protein in the treated B16-cells, while qPCR showed a 4.5 to 8.1 fold increase in gp100 (pmel17) when compared to β-actin and GAPDH reference genes, respectively (Table 4). The calculation of fold increase assumes that the reference genes remain the same with or without treatment. The discrepancy in the fold increase of gp100 transcripts as measured against GAPDH and β-actin could be due to the effect of iHsp90 on one or both of these reference genes. BIIB treatment is toxic to B-16 cells and causes the cells to enlarge and become more granular even at less than lethal doses. Since β-actin is a cytoskeletal protein it is conceivable that treatments that alter the cell structure could have an effect on this protein.

**Table 4:** Effect of the iHsp90 CCT018159 on the melanoma antigen gp100 in the mouse B-16 cell line.

Treatment^a^	µg/ml^b^	gp100 expression by Flow Cytometry (mean channel fluorescence)^c^	Change in gp100 mRNA relative to GAPDH^d^	Change in gp100 mRNA relative to β-actin^d^
Control	—	71.5 (1)	1	1
CCT018159	2.5	331.8 (4.6) (p<0.00001)	8.1 (p<0.003)	4.5 (p<0.001)

a Cells were untreated (control) or treated with the Hsp90 inhibitor CCT018159 as indicated for 3 days.

b Dose indicated is optimal dose for antigen increase

c Number represents geometric mean of intracellular staining with an antibody to gp100 of live gated cells. Number in parenthesis is fold increase relative to untreated control.

d Number represents the fold increase relative to untreated control. Fold difference is calculated as 2^-ΔΔCt^ (Materials and Methods).

p value from Student t test comparing iHsp90-treated cells to control. Data are highly significant with respect to control values for each of the comparisons shown.

#### (B) Effect of Hsp90 Inhibitor treatment on cell growth

**Figure 3:**
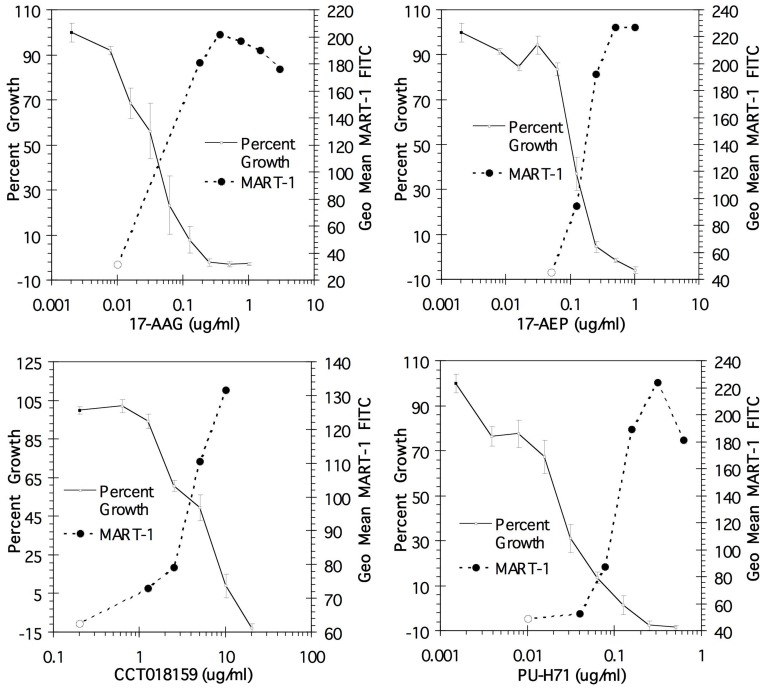
Effect of Hsp90 inhibition on MU89 growth. A WST assay was used to assess cell numbers in control and Hsp90-inhibitor treated tumors. WST levels were assayed at time zero and after 3 days. Cells were treated with the indicated Hsp90 inhibitors at the doses indicated. Percent growth was calculated as described in Methods and is plotted on the left y-axis. Data represent the average and standard deviation of triplicate wells. The level of Melan-A/MART-1 (geometric mean), as assayed by intracellular staining and flow cytometry, is shown for comparison on the right y-axis. The data for Melan-A/MART-1 staining are from one representative experiment.

The iHsp90s, 17-AAG, 17-AEP, CCT018159, and PU-H71 were tested for effects on cell growth. The toxicity of iHsp90 to melanoma cells has been reported [32]. The WST assay was used to assess the effect of Hsp90 inhibitor treatment on melanoma cell growth over a range of doses. A linear increase in growth inhibition was observed. As shown in Fig. 3, the increased levels of Melan-A/MART-1 antigen induction correlate with the decrease in cell growth. In fact, the doses required for total growth inhibition and maximal Melan-A/MART-1 induction correspond. Thus, the optimal dose for antigen expression occurs at doses of Hsp90 inhibitor that significantly inhibit growth of tumor cells.

#### (C) Hsp90 Inhibitor Treatment Kinetics

**Figure 4:**
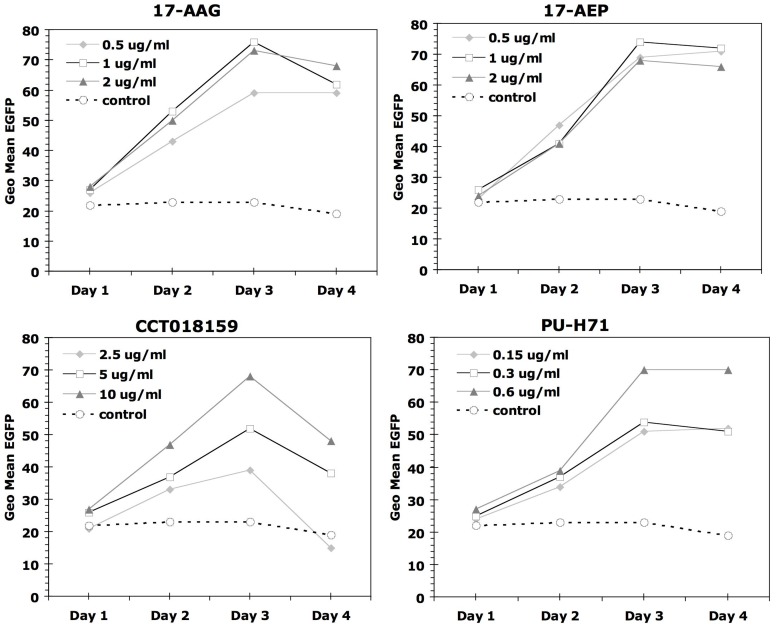
Kinetics of Melan-A/MART-1 increase. The flow cytometry data show effect of four Hsp90 inhibitors on the MU89 MART::EGFP cell line at the indicated doses as assessed over time. The same number of cells per well were plated in each well of a 24 well plate and drug was added on day zero. Each day cells were collected and assayed for that time point. Control untreated cells are shown for comparison. The data are from one representative experiment.

**Figure 5:**
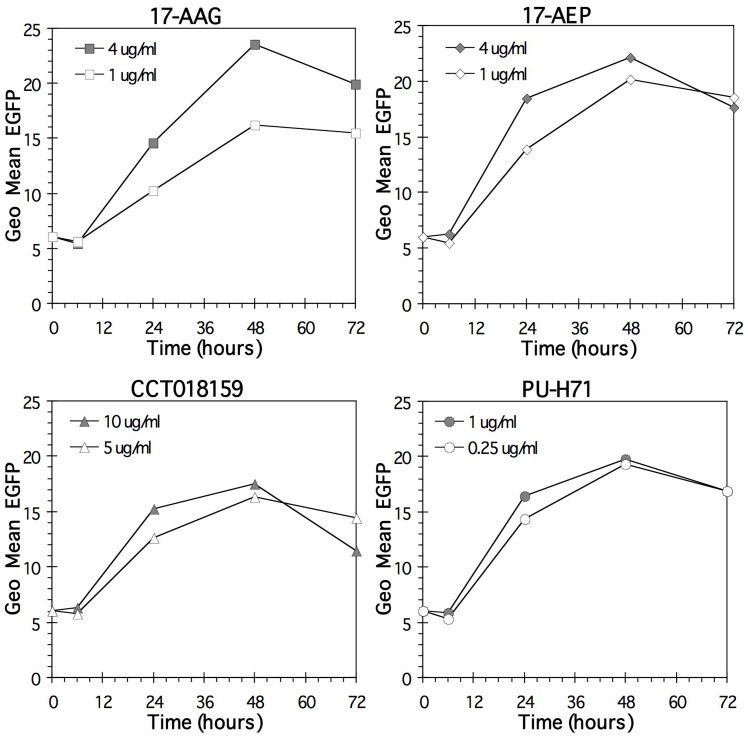
Transient exposure to HSP90 inhibitors effect on Melan-A/MART-1 promoter driven EGFP expression. In order to determine the requirement for continued Hsp90 inhibitor exposure to achieve enhanced promoter activity, the A375 MART::EGFP cell line was exposed to four different Hsp90 inhibitors for the times indicated. In each case the measurement of EGFP-fluorescence was assayed on day 3. At the times indicated, media with the Hsp90 inhibitor was removed and replaced with media without drug. The data are from one representative experiment.

We performed a kinetic analysis to determine the effect of iHsp90 on Melan-A/MART-1 promoter activity over time. Using the Melan-A/MART-1 promoter EGFP system in the melanoma cell line MU89, we showed that cells treated with 4 separate Hsp90 inhibitors (17-AAG, 17-AEP, CCT018159, and PU-H71) significantly enhanced the fluorescent reporter signal as early as 2 days (Fig. 4). Reporter activity increases steadily for the first 72 hours of Hsp90 inhibition, and then plateaus. We further assessed the need for continued presence of Hsp90 inhibitors in order for them to be effective, (whether the drug can be removed after short exposure, or whether it must it be continually present). Transient exposure of EGFP-expressing A375 cells to the Hsp90 inhibitors 17-AAG, 17-AEP, CCT018159, and PU-H71 requires a minimum of 24 hours of exposure in order to induce significant increases in Melan-A/MART-1 promoter EGFP reporter levels (Fig. 5). However, to achieve full signal enhancement, it is necessary to retain the drug for at least 48 hours, after which the effect reaches a plateau.

#### (D) Hsp90 inhibitor treatment changes level of MAPK pathway signaling

**Figure 6:**
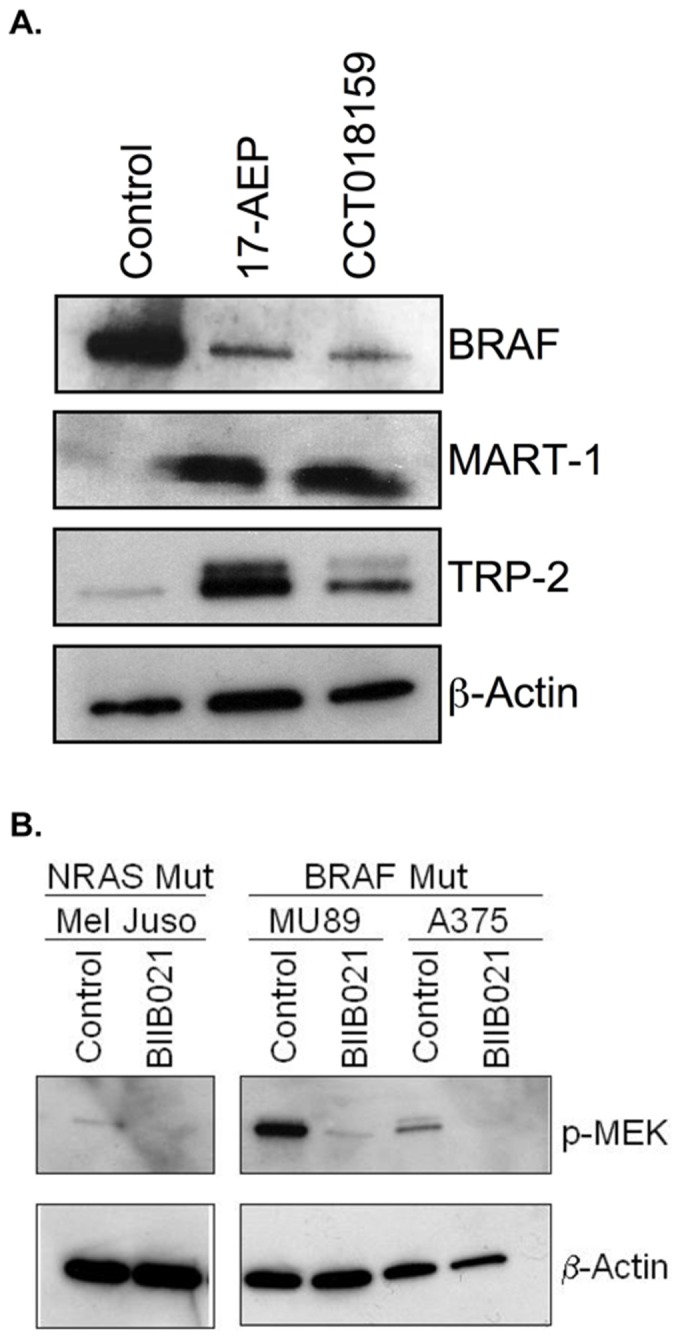
Characterization of Hsp90 inhibition of MAPK signal transduction pathway and Melanoma-Associated Antigens. A. Western blot performed on extracts of the MU89 cell line treated as indicated for three days. Extracts were probed with antibodies to BRAF, Melan-A/MART-1, TRP-2, or β-Actin in rows top to bottom as indicated. 30 µg of total protein was loaded in each lane of the gel. 1.0 µg/ml of 17-AAG, 1.0 µg/ml of 17-AEP, or 2.5 µg/ml CCT018159 were used to treat the cells. B. Protein gel electrophoresis was performed using 30 µg of total protein extracts prepared from the indicated cell lines. Cells were untreated (control) or treated with 0.15 µg/ml of BIIB021 for three days. After transfer, Western blots were probed with antibodies to phosphorylated MEK (p-MEK) or β-Actin.

To address the effect of Hsp90 inhibition on the MAPK pathway we analyzed protein levels and signaling activity (protein phosphorylation levels) of the proteins in this pathway directly. Since both BRAF and NRAS are known Hsp90 client proteins, these proteins will be destabilized when Hsp90 is inhibited. Western blots of the BRAF protein confirmed that this protein was degraded after Hsp90 inhibitor treatment (Fig. 6A; top panel). In contrast to the decreased level of BRAF in iHsp90-treated cells, the level of melanocyte antigens increased strongly (MART-1, TRP-2) while β-Actin increased slightly (Fig. 6A; lower 3 gel panels). Western blotting for phosphorylated MEK showed that signaling was blocked by iHsp90 treatment of both cells that are mutant for NRAS (and also WT BRAF), as well as WT NRAS cells that are also mutant for BRAF (Fig. 6B).

#### (E) iHsp90 treatment of melanomas increases T cell recognition

**Figure 7:**
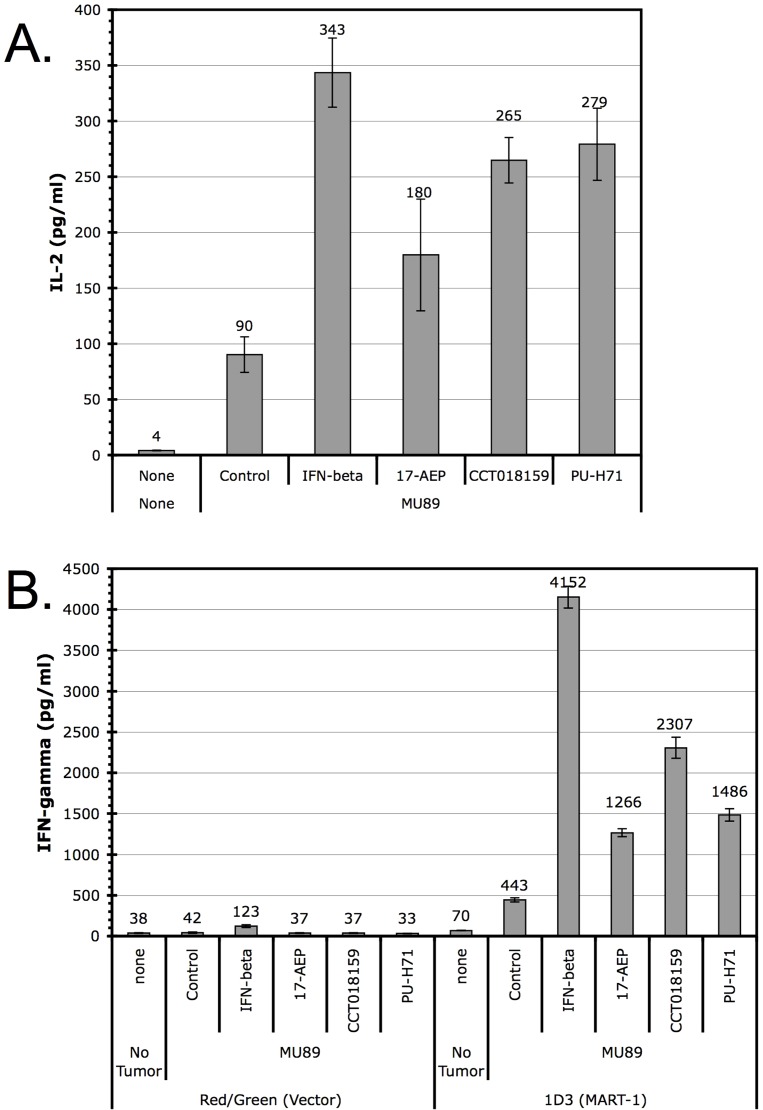
Increased T-cell recognition of Hsp90 inhibitor-treated tumor cells. A. Tumor cells were co-cultured with the Jurkat T cell line expressing the Melan-A/MART-1 specific T cell receptor and IL-2 secretion was measured by ELISA. B. Tumor cells were co-cultured with CD8+ isolated from PBLs and infected with the either a vector control or a Melan-A/MART-1 reactive TCR (about 20% positive infection determined by tetramer staining and flow cytometry). The treated MU89 tumor cells used for co-culture in both experiments (A and B) are the same. Cells were treated for three days with IFN-beta (5000 U/ml), 17-AEP (0.5 µg/ml), CCT018159 (5 µg/ml), or PU-H71 (0.15 µg/ml) for three days in a flask before being collected and counted. A 25∶10 ratio of tumor cells to T cells were mixed for co-culture (5×10^4^ of tumor cells and 2×10^4^ T cells).

We tested three iHsp90s (17-AEP, CCT and PU-H71) in a cell-based assay in which increased IL-2 secretion by responding T cells is a manifestation of tumor cell recognition [16]. Jurkat cells transduced with a TCR specific for Melan-A/MART-1 showed increased IL-2 production when co-cultured with iHsp90-treated tumors than with untreated (control) tumor cells (Fig.7A), as an indication of enhanced T cell recognition as a consequence of inhibited Hsp90 function.

The same anti-Melan-A/MART-1 specific TCR used in the Jurkat cells was also transduced into normal CD8+ Peripheral Blood Leukocyte (PBL)-derived T cells. In Fig. 7B, increased IFN-γ levels were produced by responding T cells after tumors were treated with 17-AEP, CCT or PU-H71. Tumor cells treated with iHsp90 stimulated a 3 and 5-fold increase in cytokine production by the transduced PBL when compared to the response of these PBL to untreated tumor cell controls.

## Discussion

Hsp90 has documented chaperone function that assists protein folding and prevents aggregation of non-native proteins [33]. Hsp90 is also required for the function of several oncogenes, including epidermal growth factor [34], Her-2, and BRAF (reviewed in [20]). BRAF is especially relevant in melanoma progression because of the high frequency of its activating mutations [35] and resistance to its inhibition [36]. iHsp90s have been previously reported to be potential tools in combating tumor growth, ascribed to several distinct mechanisms [37], including inhibiting both tumor cell anchorage and proliferation [38], as well as induction of apoptosis as a result of blocking NF-kappaB and Akt [39]. In a pancreatic cancer iHsp90 resulted in potent anti-tumor activity through several molecules belonging to the class of intracellular signal transduction, immune response, cell growth and maintenance [40]. Although several Hsp90 inhibitors are already being used in clinical trials [41], [42] the utility of this class of molecules in cancer therapy is still unproven [43].

As we demonstrate in this communication, iHsp90s represent a class of agents with the ability to enhance antigen expression in a variety of tumor cell lines, including a series of cell lines derived from human melanomas, a murine-derived melanoma cell line, and two human glioma cell lines. The panel of iHsp90 we tested included several compounds related to 17-AAG, but also included distinct structural entities that share only the ability to interfere with Hsp90 function. Although the most effective dose for each compound varied, each iHsp90 was active on diverse melanomas.

In addition, the data show that the enhanced antigen expression on tumors includes both differentiation antigens, (Melan-A/MART-1, gp100 and TRP2), and MHC Class I antigen. These combined increases in antigen levels lead to improved T cell recognition of treated tumor cells. As we have previously shown, several diverse compounds share the ability to enhance antigen expression by tumors, including IFN-β [10], MAPK inhibitors [11] and topoisomerases [15]. While IFN-β is not highly toxic to tumors, both iHsp90s and MAPK inhibitors are often toxic to tumors at doses that are needed to enhance antigen expression. While the stress induced by agents such as topoisomerases and iHsp90s may play a role in antigen induction, the fact that IFN-β enhances tumor antigen recognition without accompanying toxicity implies additional pathways not directly related to toxicity. As a chaperone, Hsp90 has a wide range of client proteins [41], many of which have essential roles in cellular operations. Thus, irrespective of experimental focus on specific clients, attainment of effective inhibition of Hsp90 chaperoning will most likely impair vital cellular functions as an accompanying global effect. In turn, it is not mechanistically surprising that the iHsp90 toxicity dose response parallels the dose levels required to destabilize specific client proteins associated with increased immune recognition. In any case, despite the toxicity of iHsp90s, the treated tumors are demonstrably better recognized by antigen-specific T cells. In contrast, topoisomerases are highly toxic not only to tumors, but also to T cells, making this class of drugs less satisfactory for combination immunotherapy [15], [16].

In contrast to iHsp90 impact on Hsp90 client proteins, the Western blot data show that the observed augmentation of melanocyte differentiation antigens in response to iHsp90 results from increased levels of full-length protein, a further indication that Melan-A/MART-1, gp100 and TRP-2 are not Hsp90 client proteins. These observations are in contrast to previous reports of iHsp90 drugs that lead to antigen degradation of Hsp90 client proteins, such as Her-2 [22] and EphA2 [23]. While the mechanism for antigen augmentation by iHsp90 remains to be determined, the data are consistent with transcriptional regulatory mechanisms, rather than a direct impact of iHsp90 on the antigens themselves. Not only is more intracellular protein detectable by Flow Cytometry, Western Blot and EGFP reporter signals, but the qPCR evidence also shows an increase in mRNA in iHsp90 treated cells. Taken together, these data make a cogent case for true enhanced expression of these proteins. While the list of Hsp90 client proteins is long [44], proteins mediating signal transduction and regulating transcription are frequently found [45]. Therefore, a transcriptional up-regulatory effect is both feasible and consistent with current data as the most likely mechanism underlying antigen augmentation by iHsp90.

Indeed, the impact on BRAF levels in iHsp90-treated cells indicates that MAPK-regulated pathways could be targets of iHsp90s in cells where activated mutant BRAF has been shown to at least transiently down-modulate antigen expression [11]. However, it is clear that BRAF WT cells also respond to iHsp90s with increased antigen expression, as cells expressing only NRAS mutations, as well as cells that are WT for both of these genes, (such as the human gliomas [46]) and the murine melanoma) all are susceptible to iHsp90 antigen induction.

The kinetic studies indicate that it takes at least 2 days of continuous iHsp90 treatment to achieve optimal antigen induction. This result too is consistent with antigen induction resulting from indirect regulatory events that take time to develop, rather than through alternative mechanisms where direct interactions of antigens with Hsp90 are implicated.

It is important to note that the role of iHsp90 in immune responses is complicated by countervailing forces. For example, it has been shown that Hsp90 inhibition causes decreased cell surface expression of co-stimulatory molecules, leading to inhibition of dendritic cell function [47]. Moreover, Hsp90 is required for MHC- Class II antigen presentation by B-cells [48]. Hsp90 is also required for Type I and II interferon signaling [49], so that its inhibition may result in impaired responses to these important anti-cancer cytokines, and reduced expression of both Class I and Class II antigens needed for CD8+ and CD4+ T cell receptor specificity. Preliminary data indicate that while Hsp90 inhibitors do indeed prevent interferon signaling when the two drugs are added simultaneously. Yet if tumor cells are pre-treated with interferon, and subsequently Hsp90 inhibitors are added, there may be synergistic effect of these interferon-stimulated cells, indicating that timed pulsed treatments could indeed provide enhanced antigen expression (manuscript in preparation).

However, there is also evidence that iHsp90s can enhance anti- tumor immunity. The degradation of EphA2 [23], and Her-2 [22], leads to enhanced recognition by CTL, probably as a result of increased turnover and expression on MHC Class I antigens. The augmented recognition of tumor cells we describe is a result of a true increase in tumor associated antigen expression. For these studies, TCR-transfected tumor cells were used for screening purposes, and the same TCR was used to transfect freshly-isolated CD8+ PBL. While these cells are not identical to naturally-occurring anti-tumor T cells, the TCR used was derived from a cloned T cell derived from Tumor Infiltrating Lymphocytes [6] with known anti-Melan-A/MART-1 specific cytotoxic activity [4]. The ability to use these defined effector cells adds consistency to our assays, and also provides a parallel to the growing use of CAR-expressing T cells for immunotherapy [5].

One should be cautious in pointing out that the increased immune reactivity against tumor cells could also lead to increase auto-reactivity against normal cells sharing differentiation antigens, such as Melan-A/MART-1 and gp100. Indeed, it has been noted in both animals and human clinical studies that improved efficacy of immunotherapy is often accompanied by “off-target” destruction of melanocytes [50], leading to vitiligo. Retinitis has also been observed [51]. While of concern, it is fortunate that these toxicities have, in general, been tolerable, and treatable [51]. While some serious auto-immune toxicities have been reported, particularly in patients being treated with anti-CTLA-4 antibodies [52], in most cases the risk of autoimmune complications have been outweighed by the need to treat advanced cancers.

In summary, we present data that iHsp90s represent a class of drugs with the ability to enhance T cell recognition of tumors by inducing increased expression of both differentiation antigens and MHC Class I molecules. Moreover, iHsp90s are functionally compatible with T cell anti-tumor immunity, and in murine models of tumors have shown efficacy in EphA2-directed therapy [23]. While this murine immunotherapy model targeted an Hsp90 client protein, in contrast to the antigen induction we have observed, the results emphasize that iHsp90s are fully compatible with T cell anti-tumor immunity, and thus could be useful in combination immunotherapy by targeting tumors that can otherwise escape immune destruction through low expression of T cell recognition molecules, including both differentiation antigens and presentation-restricting MHC molecules.

## References

[1] Pandolfi F, Cianci R, Lolli S, Dunn IS, Newton EE, et al (2008) Strategies to overcome obstacles to successful immunotherapy of melanoma. Int J Immunopathol Pharmacol 21:493–500. 18831916 10.1177/039463200802100302 PMC2915778

[2] Fox BA, Schendel DJ, Butterfield LH, Aamdal S, Allison JP, et al (2011) Defining the critical hurdles in cancer immunotherapy. J Transl Med 9:214. 22168571 10.1186/1479-5876-9-214 PMC3338100

[3] Restifo NP, Dudley ME, Rosenberg SA (2012) Adoptive immunotherapy for cancer: harnessing the T cell response. Nat Rev Immunol 12:269–281. 22437939 10.1038/nri3191 PMC6292222

[4] Hishii M, Kurnick JT, Ramirez-Montagut T, Pandolfi F (1999) Studies of the mechanism of cytolysis by tumour-infiltrating lymphocytes. Clin Exp Immunol 116:388–394. 10361224 10.1046/j.1365-2249.1999.00879.x PMC1905310

[5] Maus MV, June CH (2014) CARTs on the Road for Myeloma. Clin Cancer Res 20:3899–3901. 24919574 10.1158/1078-0432.CCR-14-0721 PMC4119563

[6] Hishii M, Andrews D, Boyle LA, Wong JT, Pandolfi F, et al (1997) In vivo accumulation of the same anti-melanoma T cell clone in two different metastatic sites. Proc Natl Acad Sci U S A 94:1378–1383. 9037061 10.1073/pnas.94.4.1378 PMC19799

[7] Durda PJ, Dunn IS, Rose LB, Butera D, Benson EM, et al (2003) Induction of "antigen silencing" in melanomas by oncostatin M: down-modulation of melanocyte antigen expression. Mol Cancer Res 1:411–419. 12692260

[8] Coussens LM, Zitvoge L, Palucka AK (2013) Neutralizing Tumor-Promoting Chronic Inflammation: A Magic Bullet? Science 339:286–291. 23329041 10.1126/science.1232227 PMC3591506

[9] Peggs KS, Quezada SA, Chambers CA, Korman AJ, Allison JP (2009) Blockade of CTLA-4 on both effector and regulatory T cell compartments contributes to the antitumor activity of anti-CTLA-4 antibodies. J Exp Med 206:1717–1725. 19581407 10.1084/jem.20082492 PMC2722174

[10] Dunn IS, Haggerty TJ, Kono M, Durda PJ, Butera D, et al (2007) Enhancement of human melanoma antigen expression by IFN-beta. J Immunol 179:2134–2142. 17675472 10.4049/jimmunol.179.4.2134

[11] Kono M, Dunn IS, Durda PJ, Butera D, Rose LB, et al (2006) Role of the mitogen-activated protein kinase signaling pathway in the regulation of human melanocytic antigen expression. Mol Cancer Res 4:779–792. 17050671 10.1158/1541-7786.MCR-06-0077

[12] Kurnick JT, Ramirez-Montagut T, Boyle LA, Andrews DM, Pandolfi F, et al (2001) A novel autocrine pathway of tumor escape from immune recognition: melanoma cell lines produce a soluble protein that diminishes expression of the gene encoding the melanocyte lineage melan-A/MART-1 antigen through down-modulation of its promoter. J Immunol 167:1204–1211. 11466335 10.4049/jimmunol.167.3.1204

[13] Kurnick JT, Pandolfi F, Pawelec G (2013) Current Challenges in Immunology. Internatl Trends in Immunity 1:5–9.

[14] Dunn GP, Bruce AT, Sheehan KC, Shankaran V, Uppaluri R, et al (2005) A critical function for type I interferons in cancer immunoediting. Nat Immunol 6:722–729. 15951814 10.1038/ni1213

[15] Haggerty TJ, Dunn IS, Rose LB, Newton EE, Martin S, et al (2011) Topoisomerase inhibitors modulate expression of melanocytic antigens and enhance T cell recognition of tumor cells. Cancer Immunol Immunother 60:133–144. 21052994 10.1007/s00262-010-0926-x PMC3108190

[16] Haggerty TJ, Dunn IS, Rose LB, Newton EE, Kurnick JT (2012) A Screening Assay to Identify Agents That Enhance T Cell Recognition of Human Melanomas. Assay Drug Dev Technol 10:187–201. 22085019 10.1089/adt.2011.0379 PMC3323935

[17] Grbovic OM, Basso AD, Sawai A, Ye Q, Friedlander P, et al (2006) V600E B-Raf requires the Hsp90 chaperone for stability and is degraded in response to Hsp90 inhibitors. Proc Natl Acad Sci U S A 103:57–62. 16371460 10.1073/pnas.0609973103 PMC1325013

[18] Whitesell L, Lindquist SL (2005) HSP90 and the chaperoning of cancer. Nat Rev Cancer 5:761–772. 16175177 10.1038/nrc1716

[19] Shipp C, Weide B, Derhovanessian E, Pawelec G (2013) Hsps are up-regulated in melanoma tissue and correlate with patient clinical parameters. Cell Stress and Chaperones 18:145–154. 22872370 10.1007/s12192-012-0363-1 PMC3581625

[20] Porter JR, Fritz CC, Depew KM (2010) Discovery and development of Hsp90 inhibitors: a promising pathway for cancer therapy. Curr Opin Chem Biol 14:412–420. 20409745 10.1016/j.cbpa.2010.03.019

[21] Solit DB, Osman I, Polsky D, Panageas KS, Daud A, et al (2008) Phase II trial of 17-allylamino-17-demethoxygeldanamycin in patients with metastatic melanoma. Clin Cancer Res 14:8302–8307. 19088048 10.1158/1078-0432.CCR-08-1002 PMC2629404

[22] Castilleja A, Ward NE, O'Brian CA, Swearingen B 2nd, Swan E, et al (2001) Accelerated HER-2 degradation enhances ovarian tumor recognition by CTL. Implications for tumor immunogenicity. Mol Cell Biochem 217:21–33. 11269662 10.1023/a:1007267814251

[23] Rao A, Taylor J, Chi-Sabins N, Kawabe M, Gooding W, et al (2012) Combination therapy with HSP90 inhibitor 17-DMAG reconditions the tumor microenvironment to improve recruitment of therapeutic T cells. Cancer Res 72:3196–3206. 22552283 10.1158/0008-5472.CAN-12-0538 PMC3389149

[24] van Elsas A, Hurwitz AA, Allison JP (1999) Combination immunotherapy of B16 melanoma using anti-cytotoxic T lymphocyte-associated antigen 4 (CTLA-4) and granulocyte/macrophage colony-stimulating factor (GM-CSF)-producing vaccines induces rejection of subcutaneous and metastatic tumors accompanied by autoimmune depigmentation. J Exp Med 190:355–366. 10430624 10.1084/jem.190.3.355 PMC2195583

[25] Wang Y, Zhu W, Levy D (2006) Nuclear and cytoplasmic mRNA quantification by SYBR green based real-time RT-PCR. Methods 39:356–362. 16893657 10.1016/j.ymeth.2006.06.010

[26] Schmittgen T, Zakrajsek B, Mills A, Gorn V, Singer M, et al (2000) Quantitative Reverse Transcription–Polymerase Chain Reaction to Study mRNA Decay: Comparison of Endpoint and Real-Time Methods. Analytical Biochemistry 285:194–204. 11017702 10.1006/abio.2000.4753

[27] da Rocha Dias S, Friedlos F, Light Y, Springer C, Workman P, et al (2005) Activated B-RAF is an Hsp90 client protein that is targeted by the anticancer drug 17-allylamino-17-demethoxygeldanamycin. Cancer Res 65:10686–10691. 16322212 10.1158/0008-5472.CAN-05-2632

[28] Davies H, Bignell GR, Cox C, Stephens P, Edkins S, et al (2002) Mutations of the BRAF gene in human cancer. Nature 417:949–954. 12068308 10.1038/nature00766

[29] Taldone T, Chiosis G (2009) Purine-scaffold Hsp90 inhibitors. Curr Top Med Chem 9:1436–1446. 19860732 10.2174/156802609789895737 PMC4699796

[30] Donnelly A, Blagg BS (2008) Novobiocin and additional inhibitors of the Hsp90 C-terminal nucleotide-binding pocket. Curr Med Chem 15:2702–2717. 18991631 10.2174/092986708786242895 PMC2729083

[31] Zhang T, Li Y, Yu Y, Zou P, Jiang Y, et al (2009) Characterization of celastrol to inhibit hsp90 and cdc37 interaction. J Biol Chem 284:35381–35389. 19858214 10.1074/jbc.M109.051532 PMC2790967

[32] Pacey S, Gore M, Chao D, Banerji U, Larkin J, et al (2012) A Phase II trial of 17-allylamino, 17-demethoxygeldanamycin (17-AAG, tanespimycin) in patients with metastatic melanoma. Invest New Drugs 30:341–349. 20683637 10.1007/s10637-010-9493-4

[33] Wiech H, Buchner J, Zimmermann R, Jakob U (1992) Hsp90 chaperones protein folding in vitro. Nature 358:169–170. 1614549 10.1038/358169a0

[34] Adachi S, Yasuda I, Nakashima M, Yamauchi T, Yamauchi J, et al (2010) HSP90 inhibitors induce desensitization of EGF receptor via p38 MAPK-mediated phosphorylation at Ser1046/1047 in human pancreatic cancer cells. Oncol Rep 23:1709–1714. 20428829 10.3892/or_00000815

[35] Montagut C, Sharma SV, Shioda T, McDermott U, Ulman M, et al (2008) Elevated CRAF as a potential mechanism of acquired resistance to BRAF inhibition in melanoma. Cancer Res 68:4853–4861. 18559533 10.1158/0008-5472.CAN-07-6787 PMC2692356

[36] Solit DB, Rosen N (2011) Resistance to BRAF inhibition in melanomas. N Engl J Med 364:772–774. 21345109 10.1056/NEJMcibr1013704

[37] Li Y, Zhang T, Schwartz SJ, Sun D (2009) New developments in Hsp90 inhibitors as anti-cancer therapeutics: mechanisms, clinical perspective and more potential. Drug Resist Updat 12:17–27. 19179103 10.1016/j.drup.2008.12.002 PMC2692088

[38] Mehta PP, Kung PP, Yamazaki S, Walls M, Shen A, et al (2010) A novel class of specific Hsp90 small molecule inhibitors demonstrate in vitro and in vivo anti-tumor activity in human melanoma cells. Cancer Lett 300:30–39. 10.1016/j.canlet.2010.09.002 20926183

[39] Bai L, Xu S, Chen W, Li Z, Wang X, et al (2010) Blocking NF-kappaB and Akt by Hsp90 inhibition sensitizes Smac mimetic compound 3-induced extrinsic apoptosis pathway and results in synergistic cancer cell death. Apoptosis 16:45–54. 10.1007/s10495-010-0542-4 PMC3079540 20862547

[40] Song D, Chaerkady R, Tan AC, Garcia-Garcia E, Nalli A, et al (2008) Antitumor activity and molecular effects of the novel heat shock protein 90 inhibitor, IPI-504, in pancreatic cancer. Mol Cancer Ther 7:3275–3284. 18852131 10.1158/1535-7163.MCT-08-0508

[41] Modi S, Stopeck A, Linden H, Solit D, Chandarlapaty S, et al (2011) HSP90 inhibition is effective in breast cancer: a phase II trial of tanespimycin (17-AAG) plus trastuzumab in patients with HER2-positive metastatic breast cancer progressing on trastuzumab. Clin Cancer Res 17:5132–5139. 21558407 10.1158/1078-0432.CCR-11-0072

[42] Trepel J, Mollapour M, Giaccone G, Neckers L (2010) Targeting the dynamic HSP90 complex in cancer. Nat Rev Cancer 10:537–549. 20651736 10.1038/nrc2887 PMC6778733

[43] Banerji U, Affolter A, Judson I, Marais R, Workman P (2008) BRAF and NRAS mutations in melanoma: potential relationships to clinical response to HSP90 inhibitors. Mol Cancer Ther 7:737–739. 18375819 10.1158/1535-7163.MCT-08-0145

[44] Echeverria PC, Bernthaler A, Dupuis P, Mayer B, Picard D (2011) An interaction network predicted from public data as a discovery tool: application to the hsp90 molecular chaperone machine. PLoS One 6:e26044. 22022502 10.1371/journal.pone.0026044 PMC3195953

[45] Taipale M, Jarosz DF, Lindquist S (2010) HSP90 at the hub of protein homeostasis: emerging mechanistic insights. Nat Rev Mol Cell Biol 11:515–528. 20531426 10.1038/nrm2918

[46] Schindler G, Capper D, Meyer J, Janzarik W, Omran H, et al (2011) Analysis of BRAF V600E mutation in 1,320 nervous system tumors reveals high mutation frequencies in pleomorphic xanthoastrocytoma, ganglioglioma and extra-cerebellar pilocytic astrocytoma. Acta Neuropathol 121:397–405. 21274720 10.1007/s00401-011-0802-6

[47] Bae J, Mitsiades C, Tai YT, Bertheau R, Shammas M, et al (2007) Phenotypic and functional effects of heat shock protein 90 inhibition on dendritic cell. J Immunol 178:7730–7737. 17548610 10.4049/jimmunol.178.12.7730

[48] Houlihan JL, Metzler JJ, Blum JS (2009) HSP90alpha and HSP90beta isoforms selectively modulate MHC class II antigen presentation in B cells. J Immunol 182:7451–7458. 19494268 10.4049/jimmunol.0804296 PMC2714911

[49] Shang L, Tomasi TB (2006) The heat shock protein 90-CDC37 chaperone complex is required for signaling by types I and II interferons. J Biol Chem 281:1876–1884. 16280321 10.1074/jbc.M509901200

[50] Hurwitz AA, Ji Q (2004) Autoimmune depigmentation following sensitization to melanoma antigens. Methods Mol Med 102:421–427. 15286398 10.1385/1-59259-805-6:421

[51] Yeh S, Karne NK, Kerkar SP, Heller CK, Palmer DC, et al (2009) Ocular and systemic autoimmunity after successful tumor-infiltrating lymphocyte immunotherapy for recurrent, metastatic melanoma. Ophthalmology 116:981–989. 19410956 10.1016/j.ophtha.2008.12.004 PMC2715843

[52] Tarhini AA, Edington H, Butterfield LH, Lin Y, Shuai Y, et al (2014) Immune monitoring of the circulation and the tumor microenvironment in patients with regionally advanced melanoma receiving neoadjuvant ipilimumab. PLoS One 9:e87705. 24498358 10.1371/journal.pone.0087705 PMC3912016

[53] Schulte TW, Neckers LM (1998) The benzoquinone ansamycin 17-allylamino-17-demethoxygeldanamycin binds to HSP90 and shares important biologic activities with geldanamycin. Cancer Chemother Pharmacol 42:273–279. 9744771 10.1007/s002800050817

[54] Tian ZQ, Liu Y, Zhang D, Wang Z, Dong SD, et al (2004) Synthesis and biological activities of novel 17-aminogeldanamycin derivatives. Bioorg Med Chem 12:5317–5329. 15388159 10.1016/j.bmc.2004.07.053

[55] Sharp SY, Boxall K, Rowlands M, Prodromou C, Roe SM, et al (2007) In vitro biological characterization of a novel, synthetic diaryl pyrazole resorcinol class of heat shock protein 90 inhibitors. Cancer Res 67:2206–2216. 17332351 10.1158/0008-5472.CAN-06-3473

[56] Sharma SV, Agatsuma T, Nakano H (1998) Targeting of the protein chaperone, HSP90, by the transformation suppressing agent, radicicol. Oncogene 16:2639–2645. 9632140 10.1038/sj.onc.1201790

[57] Lundgren K, Zhang H, Brekken J, Huser N, Powell RE, et al (2009) BIIB021, an orally available, fully synthetic small-molecule inhibitor of the heat shock protein Hsp90. Mol Cancer Ther 8:921–929. 19372565 10.1158/1535-7163.MCT-08-0758

[58] Brough PA, Aherne W, Barril X, Borgognoni J, Boxall K, et al (2008) 4,5-diarylisoxazole Hsp90 chaperone inhibitors: potential therapeutic agents for the treatment of cancer. J Med Chem 51:196–218. 18020435 10.1021/jm701018h

[59] Brandt GE, Schmidt MD, Prisinzano TE, Blagg BS (2008) Gedunin, a novel hsp90 inhibitor: semisynthesis of derivatives and preliminary structure-activity relationships. J Med Chem 51:6495–6502. 18816111 10.1021/jm8007486 PMC2850591

